# Towards an ontological representation of morbidity and mortality in Description Logics

**DOI:** 10.1186/2041-1480-3-S2-S7

**Published:** 2012-09-21

**Authors:** Filipe Santana, Fred Freitas, Roberta Fernandes, Zulma Medeiros, Daniel Schober

**Affiliations:** 1 Informatics Center, Federal University of Pernambuco (CIn/UFPE), Recife, Pernambuco, 50.740-560, Brazil; 2 Parasitology Department, Aggeu Magalhães Research Center, Oswaldo Cruz Foundation, (CPqAM/Fiocruz), Recife, Pernambuco, 50.670-420, Brazil; 3 Pathology Department, Institute of Biological Sciences, University of Pernambuco, Recife, Pernambuco, 50.100-130, Brazil; 4 Institute of Medical Biometry and Medical Informatics (IMBI), University Medical Center, Freiburg, 79104, Germany

## Abstract

****Background**:**

Despite the high coverage of biomedical ontologies, very few sound definitions of death can be found. Nevertheless, this concept has its relevance in epidemiology, such as for data integration within mortality notification systems. We here introduce an ontological representation of the complex biological qualities and processes that inhere in organisms transitioning from life to death. We further characterize them by causal processes and their temporal borders.

****Results**:**

Several representational difficulties were faced, mainly regarding kinds of processes with blurred or fiat borders that change their type in a continuous rather than discrete mode. Examples of such hard to grasp concepts are life, death and its relationships with injuries and diseases. We illustrate an iterative optimization of definitions within four versions of the ontology, so as to stress the typical problems encountered in representing complex biological processes. We point out possible solutions for representing concepts related to biological life cycles, preserving identity of participating individuals, i.e. for a patient in transition from life to death. This solution however required the use of extended description logics not yet supported by tools. We also focus on the interdependencies and need to change further parts if one part is changed.

****Conclusion**:**

The axiomatic definition of mortality we introduce allows the description of biologic processes related to the transition from healthy to diseased or injured, and up to a final death state. Exploiting such definitions embedded into descriptions of pathogen transmissions by arthropod vectors, the complete sequence of infection and disease processes can be described, starting from the inoculation of a pathogen by a vector, until the death of an individual, preserving the identity of the patient.

## Introduction

With the growing need to cope with large-scale biomedical data, researchers have been relying on ontologies to ensure a shared and computer-interpretable meaning of linguistic terms describing such data, fostering intelligent information integration and interoperability [1]. Indeed, more than 250 ontologies (December 26th 2011) are available in the BioPortal ontology library [2].

Despite many efforts devoted to the development of genomics and metabolomics ontologies, often motivated by the prototypical Gene Ontology [3], few are focusing also on patient and disease centered data. This is required, for instance, in epidemiology to study the dynamics of diseases, and to define health policies for epidemiological surveillance. Morbidity databases, such as the National Morbidity Notification Information System in Brazil (SINAN) [4], are used as the main sources for epidemiological disease surveillance, prevention and control.

Furthermore, mortality databases describing the cause of death are of interest to the World Health Organization (WHO) enabling the production of local or global health-related statistics. In Brazil, this data is stored in the national Brazilian Mortality System (SIM) [5], and grouped by the primary causes of death.

If the goal is to leverage synergies resulting from querying and comparing the two databases at the same time, ontologies can play an important role by enabling a common communication channel needed to ensure semantic interoperability. Rendering the separately maintained mortality and morbidity databases accessible via a common ontology allows for synergistic data exploitation, i.e. use of contextual enrichment, consistency checks and reasoning at schema and data level [6].

The purpose of the current study is to ontologically formalize foundational disease processes and other lifecycle related processes as occurring in the mentioned data sources to expand the Neglected Tropical Disease Ontology (NTDO) [7,8] and ultimately to use NTDO for integrated querying of the Brazilian mortality and morbidity databases.

Ontologies, from a formal point of view, intend to describe a consensus on the nature of entities in a given scientific domain, independently of linguistic variation of the terms used in human communication. Accordingly, formal ontologies are expressed by means of a formal semantics, like Description Logics (DL) [1], nowadays generally using the World Wide Web Consortium (W3C) recommended exchange syntax Web Ontology Language (OWL) [9].

When trying to integrate heterogeneous databases, such as SIM and SINAN, many interesting problems arose. For instance, we observed that the identifiers in both databases do not follow strict rules so as to prevent misidentification and to leverage data integration. This syntactical problem is usually addressed by algorithms that compute cumulative evidence [10] from other pieces of the registers to decide for a matching, i.e. comparing other data than the proper identification of an individual (e.g. mother's name, birth date, among others).

However, a more interesting semantic integration problem occurred while querying the two databases together: an individual may happen to die due to a certain disease, but instead of reporting the trigger event that ultimately lead to death, i.e. a particular disorder like Chagas disease, a secondary cause, i.e. something else related to the disease like heart attack is reported as the primary cause and stored in the database. This heart complication in a Chagas patient is a frequent secondary effect of the primary cause, the Chagas infection, which should be tracked in the databases like this in order to prevent false epidemiologic measures.

The above requirement leads us to expand NTDO with classes allowing for such granular distinctions and a sound ontological representation of death. Many subtle aspects hamper a precise definition in this case, i.e. a) the conditions in which an individual is considered dead, and b) the ontological problem of preserving identity of an individual when transitioning from a living to a dead organism in different stages of disintegration.

In order to support the integration and verification of morbidity and mortality data in the SINAN and SIM databases, we here present an ontological representation of death. As an example of iterative modeling, we outline four successive versions for representing mortality and discuss representational problems or the complexity of reasoning arising from each. We conclude by briefly describing what can be done with the mortality representation and the next steps of the NTDO project.

## Methods

### Representational principles

NTDO [8] leverages classes and relations provided by the upper level ontology BioTop [11], specializing it downwards to the required leaf node granularity. Additional classes and relations for representing time intervals and their boundaries were imported from the General Formal Ontology (GFO) [12,13].

NTDO was based in established ontology construction guidelines [14], which suggested the untangling of asserted graphs into disjoint orthogonal axes, letting a DL reasoner maintain the tangled poly-hierarchy. Naming conventions provided by [15] were applied consistently.

### Representation language and semantics

NTDO has been built using the Ontology Web Language 2 (OWL2), which is the World Wide Web Consortium (W3C) [9] recommended syntax, extended with the agreement operator (≐) [1]. The semantics of OWL 2 is based on Description Logics (DL) semantics [1], and the for an interpretation *I *is as follows. If **f **and **g **are properties or role chains, an OWL 2 interpretation *I *is extended with the following interpretation function:

$${\left(f\dot{=}g\right)}^{I}=\{a\in \Delta^{I}|\exists b.\left(a,b\right)\in f^{I}\wedge \left(a,g\right)\in g^{I}\}$$

where Δ*^I ^*is the domain of the interpretation.

For instance, if we want to describe all male children named with his father name as first name, the class of people who has the same name as his father can be described as: he is Human and** hasFirstName **≐ **hasChild **◦ **hasFirstName**. It means that the first name of the child must be the same as his father's name.

The mortality representation within NTDO was edited via the ontology editor Protégé v.4.1 using the embedded reasoner HermiT [16] for classification in most steps. Inference could not be performed over agreements since OWL2, and so HermiT, is not able to handle it.

### Knowledge sources

As for the knowledge sources, apart from the literature review, other relevant sources were the morbidity and mortality systems themselves [4,5]. At some extent we grounded our definitions on the way death cases are reported to the SIM [5]. This system reports cases objectively by "primary cause of death" (e.g. a disease or an injury which triggered a chain of pathological events and led to death) and "other related causes of death" (e.g. other related disease of injury related to the death). The deaths are always identified by a forensic medicine service or the physician who was treating the subject of care for a disease, or injury, leading to the death.

As morbidity and mortality databases do contain homologous entries referring to the same subject of care, the primary cause of death in the mortality database can be correlated with the disease entry of the same subject of care in the morbidity database, postulating a causal relationship; i.e. the tracked primary death cause might be a secondary symptom of the progressing disease.

## Results

In this section, we describe our ontological representation of mortality. It assumes a disease/injury to be the primary cause of death and is necessary to describe both temporarily extended and instantaneous processes in health care relevant life cycle stages, starting with the transmission of a pathogen, over the disease as a pathological process, and finally ending in the process of dying. In the next subsections, we will provide DL definitions for all important parts of our mortality model, and represent complex issues encountered and how they were solved in our model.

### Representing injury and death

**Figure 1:**
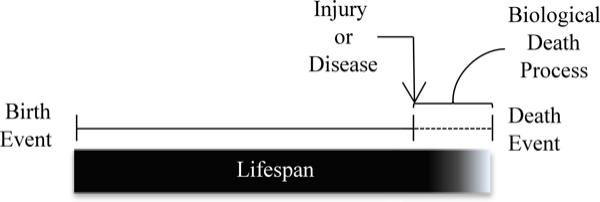
**The typical life cycle of an organism**. This figure includes the main processes and events from conception to death.

Our representation of death is based on the generalized lifecycle displayed in Figure 1. Taking the birth as the starting point, the lifespan of an individual organism continues until its death. However the lifespan may overlap at the end with the beginning of a biological death process, the one which will ultimately lead to death. This definition is grounded on the fact that several factors can simultaneously influence the lifetime of an individual organisms and provoke its death, such as accidents or illnesses.

Despite being a simplification only introduced for the easy handle of statistics, mortality registries worldwide store mainly the primary cause of death. In our ontology we follow this simplification, although extending it to accommodate multiple causes would be a quite straightforward process without additional computational costs for inferences.

At a given moment an individual organism can acquire a certain disease, e.g. dengue fever, which may cause premature death, depending on the circumstances. In medical terms, a disease cause is a function of the physiological state of the individual. There can be a causal link from a disease and its symptoms to a later death process.

Assuming all data is available, it should be possible to describe and trace the sequence of causally induced - and at times overlapping - pathologic processes which affect the life of that organism, from birth to death. Some of them may damage the organisms' overall physiological state to such an extent, that they directly initiate a process of physiological death, leading to death itself. This sequence of processes is sometimes evidenced by the records of an individual when the cause of death was previously registered in a morbidity system, i.e. the primary cause of death was already known.

Our definition of the 'Birth' process is based on the description of "live birth" provided by the Brazilian Institute of Geography and Statistics (IBGE) [17]. It corresponds to the complete expulsion or extraction of a product generated by the maternal body after conception, which after separated from the maternal body, breathes or exhibits some other vital signs, e.g. heartbeat, voluntary muscle contraction, umbilical cord contraction, regardless of the cord being cut or not, and whether or not the placenta was expelled. Conversely, "death" as a state means absence of brain functions and cessation of all biological functions, inherent to the human body [18].

However, there are major difficulties related to the accurate representation of the processes that make an individual die:

• Complexity is an issue, as the causal nature, which can be quite indirect at times with many unknown factors as comorbidities and interlaced parallel influences converging ultimately into a death process;

• Another issue is relating sequences of processes and time, with a precise description of where and when each process took place, when it started and where its boundaries are.

Nevertheless, this exact information is probably not important at all if the aim of the proposed model is to deal with mortality data. Instead what is usually known and found in the databases is the knowledge of what is the sequence of typical signs and symptoms of a disease, because the time constraints involving them, e.g. during tuberculosis, a cough with secretion follows a pulmonary infection, can be checked in morbidity and mortality notifications. For stating a death record in a mortality notification database, viz. SIM, a physician certifies one underlying primary cause of death and sometimes one or more secondary causes. The ontology should support these two descriptions.

We also assume the notion of instantaneous processes available in BioTop as equivalent to events provided in GFO, which makes the subject of care exhibit a certain behavior which is linked, causally or not, to some processes [13].

### Representational challenges of the mortality model

Next, we present the main challenges related to the representation and the logical axioms characterizing and solving these challenges. To allow the reader to follow our lines of reasoning we explain four successive versions of definitions for the core entities, demonstrating our iterative optimization approach and the evolution of the model to a final proposal.

The two major challenges encountered in creating a coherent representation for a mortality process were the preservation of the identity of related individuals by setting cardinalities, and the rendering of the resulting ontology in a decidable DL. Each of these items is discussed in the consecutive versions until we arrive at a satisfactory model.

### Version 1: Introducing the death representation

Our initial naïve definition of death was:

(1) $$\begin{array}{l} DeathEvent\;\text{equivalentTo}\;InstantaneousProcess\\ \;\;\;\text{and}\;(\mathrm{hasPatient}\;\text{some}\;DeadOrganism)\\ \;\;\;\text{and}\;\left(\mathrm{processualPartOf}\;\text{some}\;BiologicalDeathProcess\right)\\ \;\;\;\text{and}\;\left(\mathrm{hasInstant}\;\text{some}\;PointInTime\right) \end{array}$$

indicating that a death event is an instantaneous process (i.e. it happens in the very moment when the person dies) in which a dead organism is a participant. It also states that there are one or more biological processes (e.g. a disease or an injury) as part of the death event and it is not temporarily extended.

This definition lacks precision regarding how to preserve identity between the living and the dead organism, as the living individual is not specified in the axiomatic description. According to the class definition, there is no guarantee that the living and the dead body are identical, since the patients of instances of *DeathEvent *and *BiologicalDeathProcess *may not be the same.

Also, the axiom expresses no cardinality constraint, which gives rise to different interpretations, such as the possibility of more than one individual dying by the same death process. Besides, subscribing to the idea that a living organism is eventually transformed into a dead one causes further representational problems. First, our imported top level, BioTop, restricts its organism hierarchy to living ones, requiring additional class expressions to refer to dead organisms (e.g. using the relation **transformationOf**). As a consequence, a dead human is not human any more, although possessing human organs, features, etc.

Besides losing its "humanity", identity is lost too, since any classification of living beings is rigid, i.e., once an individual is an instance of a rigid class, then it ceases to be an instance only when it does not exist anymore [19]. Even if we assume that this description corresponds to a *phased sortal *[19], i.e. an entity which changes phases (from "living" to "dead"), it is not clear unless identity is be preserved, i.e. whether the ashes of a dead organism should be identified with the dead person.

### Version 2: Representing death in the temporal axis

A solution to circumvent such representational problems is simply not separately representing the entities that cause this confusion, viz. *DeadOrganism*, which, indeed, do not matter in most health-related applications. The new solution then consists in representing living organisms based on their temporal existence, limited by two time points, as described in the General Formal Ontology (GFO) [13]. Such representation employs the definition of gfo:*Chronoid*, i.e. an interval not defined as a set of points, thus implying that time is represented as a continuum, which is equivalent to biotop:*TimeInterval*.

(2) biotop:TimeIntervalequivalentTogfo:Chronoid

**Figure 2:**
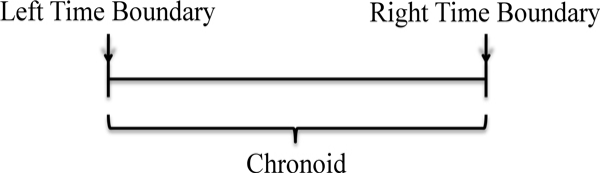
**The interval described for a gfo:*Chronoid *and its borders (gfo:*LeftTimeBoundary *and gfo:*RightTimeBoundary*), in the temporal axis**.

Every *Chronoid *has two outer boundaries, known as time limits (gfo: *TimeBoundary *or biotop:*PointInTime*) or points in time. In GFO, there are two kinds of temporal boundaries, representing the right and left limit of a temporal interval, i.e. gfo: *LeftTimeBoundary *and gfo: *RightTimeBoundary*. By definition, they cannot hold the same values in a single chronoid [13]. A schematic representation can be found in Figure 2.

For the sake of clarity, we show here the definitions of *Chronoid *and its time boundaries in

GFO:

(3) $$\begin{array}{l} \text{gfo}:Chronoid\;\text{subclassOf}\\ \;\;\;\;\left(\text{gfo}:\mathrm{hasLeftTimeBoundary}\;\text{exactly}\;\text{1}\;\text{gfo}:LeftTimeBoundary\right)\;\text{and}\\ \;\;\;\;\left(\text{gfo}:\mathrm{hasRightTimeBoundary}\;\text{exactly}\;\text{1}\;\text{gfo}:RightTimeBoundary\right) \end{array}$$

(4) $$\begin{array}{l} \text{gfo}:LeftTimeBoundary\;\text{equivalentTo}\;\text{gfo}:TimeBoundary\\ \;\;\;\;\text{and}\;\left(\text{gfo}:\mathrm{leftTimeBoundaryOf}\;\text{some}\;\text{gfo}:Chronoid\right) \end{array}$$

(5) $$\begin{array}{l} \text{gfo}:RightTimeBoundary\;\text{equivalentTo}\;\text{gfo}:TimeBoundary\\ \;\;\;\;\text{and}\;\left(\text{gfo}:\mathrm{rightTimeBoundaryOf}\;\text{some}\;\text{gfo}:Chronoid\right) \end{array}$$

When there are chronoids in sequence, the right time limit of a preceding process must be contiguous with the left of the subsequent one; the overlap representing the beginning of a new chronoid and the end of the previous.

Following the GFO and BioTop perspectives, *LivingOrganism *is represented as a *MaterialObject*. In order to define that a *LivingOrganism *can die, we need to specify that its existence is delimited, which is described in GFO.

Following this assumption, the axiom below should be included:

(6) $$\text{biotop}:LivingOrganism\;\text{subClassOf}\left(\text{gfo}:\mathrm{exists}\;\mathrm{at}\;\text{exactly}\;\text{1}\;\text{gfo}:TimeBoundary\right)$$

stating that one living organism in exists in only one time interval (its lifespan).

Aditionally, processes are projected (gfo: **projectsTo**) to *Chronoids*, i.e., they exist in the time interval represented by a *Chronoid *[13]. Establishing correspondences between GFO and BioTop to avoid mismatches in NTDO, the class gfo:*Process *must be mapped to the class biotop:*Process*.

Finally, the *DeathEvent *should be modified to replace a *DeadOrganism *by a *LivingOrganism*, as follows:

(7) $$\begin{array}{l} DeathEvent\;\text{equivalentTo}\;InstantaneousProcess\\ \;\;\;\;\text{and}\;\left(\mathrm{hasPatient}\;\text{some}\;LivingOrganism\right)\\ \;\;\;\;\text{and}\;\left(\mathrm{processualPartOf}\;\text{some}\;BiologicalDeathProcess\right)\\ \;\;\;\;\text{and}\;\left(\mathrm{hasInstant}\;\text{some}\;PointInTime\right) \end{array}$$

On the one hand, the ontological problems with the existence of *DeadOrganisms *are solved, including the identity problem, as instances of *LivingOrganism *are formed at a certain time point (gfo:*LeftTimeBoundary*) and destroyed in another (gfo:*RightTimeBoundary*). On the other hand, by definition the relationship biotop:**hasPatient **allows more than one element in the range, which can lead to the erroneous interpretation that a process of death by injury or disease happen to several people simultaneously.

Moreover, it still contains three further identity problems: (a) The one between the *DeathEvent *and the *BiologicalDeathProcess *patients; (b) the set of definitions stated up to that point neither includes the moment of death nor synchronizes it with the end of the *BiologicalDeathProcess *that led to it; and (c) the same applies to the dying *LivingOrganism*, whose *RightTimeBoundary *should coincide with both the *DeathEvent *and the end of the *BiologicalDeathProcess *converging into it. Indeed, *DeathEvent *is exactly the last temporal part of a *BiologicalDeathProcess*; this is also an issue of coherence since the opposite (a *BiologicalDeathProcess *being part of a *DeathEvent*) would mean that an instantaneous process would have as part a process related to a time interval.

### Version 3: Introducing the agreement operator to enable identity

The representation of instantaneous processes allows us to render the *RightTimeBoundary *of a *BiologicalDeathProcess *synchronous with the *DeathEvent*. For this purpose, the class iotop:*InstantaneousProcess *was used, as being a process that happens at the end of a preceding process, so as to form a process sequence, connecting the end of one process with the beginning of the next one, using the DL agreement operator (≐).

This operator is used in chains of properties to indicate that the instances to be described are connected. It is worth stressing, the difference between the two operators, ≐ and =. The former represents a coincidence in the value of two properties, or, in other words, a reference to a very same object, while the latter defines a formation rule for a property, which is usually based on property chains [1] as in the case above. In our ontology, we need, for instance, to establish that a certain process ends exactly when another starts; this is denoted by an agreement.

We now need the definition of an instant to ascribe exactly when a death takes place in order to enable the condition 'instantaneous' (ntdo: **hasInstant**) to be defined as an exact point in time. This can be reached by making instant an event that occurs solely in the right border of its process that it follows, as below:

(8) ntdo:hasInstant=gfo:projectsToogfo:hasRightTimeBoundary

It is important to disambiguate *InjuryEvent *and *DeathEvent*. For the description of an injury event, it is necessary to determine its cause and the injured subject of care. Injury causes are described here as being caused exclusively by non-biological processes. All of this is ascribed in the axiom below:

(9) $$\begin{array}{l} \text{ntdo}:InjuryEvent\;\text{subClassOf}\;\text{biotop}:InstantaneousProcess\\ \;\;\;\;\;\text{and}\;(\text{biotop}:\mathrm{causedBy}\;\text{only}\;(\text{biotop}:ProcessualEntity\\ \;\;\;\;\;\text{and}\;\left(\text{not}\;\text{biotop}:BiologicalProcessualEntity\right)))\\ \;\;\;\;\;\text{and}\;\left(\text{ntdo}:\mathrm{hasInjuredPatient}\;\text{only}\;\text{biotop}:LivingOrganism\right) \end{array}$$

Despite not being the focus of the current work, which is about deaths caused by diseases, it is necessary to distinguish pathological processes, structures, and dispositions [20]. Disorders are caused by an accident, a lesion, or a fracture and can lead to a disease. Thus, disorders follow injuries.

The new definition of a *DeathEvent *goes below:

(10) $$\begin{array}{lll} \text{ntdo}:DeathEvent\;\text{equivalentTo}\;\text{biotop}:InstantaneousProcess\\ \;\;\;\;\;\text{and}\;\left(\text{ntdo}:\mathrm{hasPatient}\;\text{some}\;\text{biotop}:LivingOrganism\right)\\ \;\;\;\;\;\text{and}\;\left(\text{ntdo}:\mathrm{precededBy}\;\text{some ntdo}:BiologicalDeathProcess\right)\\ \;\;\;\;\;\text{and}\;\left(\text{ntdo}:\mathrm{hasInstant}≐\text{ntdo}:\mathrm{precededBy}\;\text{o}\;\text{gfo}:\mathrm{hasRightTimeBoundary}\right)\\ \;\;\;\;\;\text{and}\;\left(\text{ntdo}:\mathrm{hasPrimaryDeathCause}\;\text{exactly}\;\text{1biotop}:ProcessualEntity\right)\\ \;\;\;\;\;\text{and}\;\left(\text{ntdo}:\mathrm{hasPatient}≐\text{ntdo}:\mathrm{precededBy}\;\text{o}\;\text{ntdo}:\mathrm{hasPatient}\right)\; & & \;\\ & \; & \end{array}$$

It describes which deceased organism is its patient, and which process is the primary cause of death. The agreement conditions are the more important ones. They ensure that the death occurs exactly when the *BiologicalDeathProcess *is finished (ntdo:**hasInstant **≐ ntdo:**precededBy **o gfo:**hasRightTimeBoundary**) and that a deceased person is the same who participated in the injury event that led to the death, thus retaining the identity of the subject of care (the last condition).

Finally, completing the ontological representation of mortality, the class ntdo:*BiologicalDeathProcess *was created to indicate the existence of an aggregate (summation of processes happening in parallel) of not completely known processes that occur in the dying organism, which ultimately trigger the death event. A biological death process (from disease to death) is a biological processual entity which is caused by an injury (non-biological) or biological process (but, of course, not by biological death processes themselves). It has as patient an organism and its duration is delimited:

(11) $$\begin{array}{lll} \text{ntdo}:\;BiologicalDeathProcess\;\text{subClassOf}\;\text{biotop}:BiologicalProcessualEntity\\ \;\;\;\;\text{and}\;\left(\text{biotop}:\mathrm{causedBy}\;\text{only}\;\left(\text{ntdo}:InjuryProcess\;\text{or}\;\text{biotop}:PathologicalProcess\right)\right)\\ \;\;\;\;\text{and}\;\left(\text{ntdo}:\mathrm{hasPatient}\;\text{only}\;\text{biotop}:LivingOrganism\right)\\ \;\;\;\;\text{and}\;\left(\text{gfo}:\mathrm{projectsTo}\;\text{exactly}\;\text{1}\;\text{gfo}:Chronoid\right)\; & & \;\\ & \; & \end{array}$$

This axiom addresses the processes that occur prior to the death process and after an injury or disease. As for the representation of participants (also described in *DeathEvent*), there is a need to identify the existence of one or more processes, even imperceptible or indirectly related. From the epidemiological point of view, these can only be completely defined a *posteriori*, since a previous cause (illness/injury) can only be linked to the primary cause of death in a *post mortem *analysis (by autopsy, for instance) or the statement of a physician who was taking care until the time of death. In the present ontology, from the axioms so far described, it is possible to assume a causal sequence of facts for an organism: illness/injury → biological death process → death.

The axioms formulated up to now mention only causal relationships (e.g. *InjuryProcess *or *DeathEvent*). However, this notion of causality, which is necessary for the representation, is based on the observer of the process, i.e. the physician who certified the cause of death. Taking as an example a death record in a mortality notification database, viz. SIM, a physician certifies the underlying primary cause of death and sometimes secondary ones.

In this ontology, this fact is supported by ntdo:*BiologicalDeathProcess*, because this class allows for the inclusion of more than one cause, and may be extended in the ntdo:*DeathEvent*, since we are only taking the primary cause into account here, the defining cause of death (which may not be the real one).

The presented model solved the identity problem; nevertheless a hidden problem not related to the representation but to the reasoning still remains: if agreements are not built over property chains of functional properties, then inference becomes undecidable [21].

Another subtle aspect is that biological death processes may occur due to injury and unknown causes, apart from diseases.

### Version 4: Ensuring identity with transitive object properties

**Figure 3:**
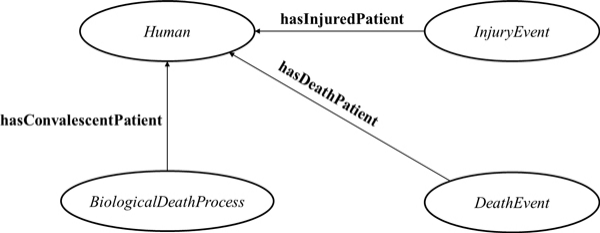
**Graphical model of an *InjuryEvent*, *DeathEvent *and *BiologicalDeathProcess *and relations (**hasInjuredPatient**, **hasDeathPatient **and **hasConvalescentPatient**), according with the processes, respective death events (InstantiousProcess) participant (e.g. *Human*)**.

The undecidability problem mentioned just above is related to the cardinality of the relationship biotop:**hasPatient**. For our purposes, this relation must be functional, i.e. each element of the domain must be mapped to at most one element of the range. In each death event, only one instance of *LivingOrganism *stands in a **hasPatient **relation to the *BiologicalDeathProcess*. Unfortunately, BioTop does not define the relation biotop:**hasPatient **as functional. Therefore, in order to meet this requirement, we created the following subproperties, all functional:

(12) $$\text{Functional}\;\left(\mathrm{hasDeathPatient},\;\mathrm{hasConvalescentPatient},\;\mathrm{hasInjuredPatient}\right)$$

indicating that an injury or death process has only one instance of *LivingOrganism *(Figure 3) as a passive participant. For instance, the functional property biotop:**hasInjuredPatient **fits perfectly to most healthcare notifications, since it refers exclusively to a single person. The property **hasConvalescentPatient **is only employed in *BiologicalDeathProcesses*, while analogously **hasDeathPatient **is used in the definition of the *DeathEvent*, as can be seen below:

(13) $$\begin{array}{lll} \text{ntdo}:DeathEvent\;\text{equivalentTo}\;\text{biotop}:InstantaneousProcess\\ \;\;\;\;\;\text{and}\;\left(\text{ntdo}:\mathrm{hasDeathPatient}\;\text{exactly}\;\text{1}\;\text{biotop}:LivingOrganism\right)\\ \;\;\;\;\;\text{and}\;(\text{ntdo}:\mathrm{hasDeathPatient}≐\text{ntdo}:\mathrm{precededBy}\;\text{o}\;\text{ntdo}:\mathrm{hasConvalescentPatient})\\ \;\;\;\;\;\text{and}\;\left(\text{ntdo}:\mathrm{precededBy}\;\text{some}\;\text{ntdo}:BiologicalDeathProcess\right)\\ \;\;\;\;\;\text{and}\;\left(\text{ntdo}:\mathrm{hasInstant}≐\text{ntdo}:\mathrm{precededBy}\;\text{o}\;\text{gfo}:\mathrm{hasRightTimeBoundary}\right)\\ \;\;\;\;\;\text{and}\;\left(\text{ntdo}:\mathrm{hasPrimaryDeathCause}\;\text{exactly}\;\text{1}\;\text{biotop}:ProcessualEntity\right)\; & & \;\\ & \; & \end{array}$$

This definition has the advantage of stressing explicitly the fact that the dead patient and the participant of a *BiologicalDeathProcess*, of two consecutive and linked processes, has the same identity.

**Figure 4:**
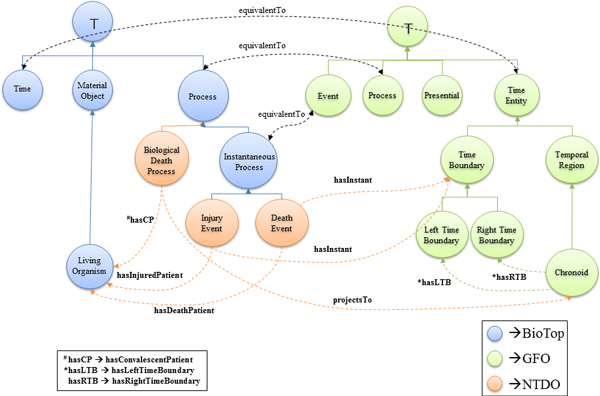
**Connections between NTDO, BioTop and GFO**. Many NTDO classes are subclasses of BioTop classes, while some GFO classes were imported from GFO.

For a better understanding, a schematic model highlighting the main classes and relations presented in the axioms for representing a death event is depicted below (Figure 4). It illustrates the classes and relations needed to model the life cycle of a biotop:*LivingOrganism*, transitioning from life to death and indicates the mappings created between BioTop and GFO, which are necessary for NTDO. In Addition, it shows some agreements required to express the temporal sequence of processes.

## Discussion

Since no ontology on mortality is available, we will compare our work with efforts that discuss mortality epistemologically. Although a related work about an ontology of death by Thomasma [22] enlists related terms and provides some connections among them, it does not provide a sound or formal definition for death. The approach to model a death event as subclass of an instantaneous process, which is applied here, is also present in [22]. For him, death can only possibly be identified by another person.

Currently, we are elaborating use cases that match morbidity and mortality databases. The ontology is being used for checking whether the notified data is correct against the constraints imposed by the complex axioms (such as impossibility of a certain disease occur in some areas) and rectifying wrong data (such as symptoms of a disease mistakenly considered as primary causes of death instead of the disease itself).

## Conclusion

In the current work, we represented complex processes, characterized by temporal marks, causality, identity preservation of the attending individuals within and the context of an objective and explicit representation of organisms transitioning from life to death. Several representational difficulties were faced, mainly regarding to the complexity of the represented entities.

Our iteratively optimized models, exemplified here by four versions of the ontology, aim at stressing the typical problems - such as preserving identity, asserting correct cardinalities and agreements among relations - encountered in representing complex biological events in description logic, as well as pointing out solutions.

The NTDO in its current status allows the description of the processes related to diseases and injuries, including their evolution that ultimately can lead to death. Using it together with other parts of NTDO, as the description of pathogen transmission by arthropod vectors, the sequence of processes can be identified, starting from the inoculation of a pathogen by a vector, until the death of an individual.

Unfortunately, the capabilities of reasoners could not be exploited, in the case the representation language they handle and the one chose for NTDO (i.e. OWL2) does not allow the usage of agreements.

Therefore, the ontology, with the current addition of mortality related contents, may serve many different purposes, such as supporting tutor systems, serving as shared vocabulary in data integration solutions, among others. The usage in data integration solutions seems to be promising, as mortality and morbidity databases contain erroneous and/or incomplete entries.

## Competing interests

We declare to have no competing interests. In the past five years, none of the authors received reimbursements, fees, funding, or salary from any organization that may, in any way, gain or lose financially from the publication of this manuscript, neither now, or in the future.

None of the authors hold stocks or shares in an organization that may gain or lose financially from the publication of this manuscript, neither now nor in the future.

None of the authors is currently applying for any patents relating to the content of the manuscript.

## Authors' contributions

FS - This author developed the main idea about the article, describing the content and creating the proposed model. During the writing, his contributions regarding the biomedical content were crucial to create the link between the biological knowledge used with the logic. He also reviewed most part of the manuscript.

FF - This author developed the main idea about the article, describing the content and creating the proposed model. During the writing, his contributions regarding the biomedical content were crucial to create the link between the logics content with the biological knowledge used. He also reviewed most part of the manuscript.

RF - This author developed the ideas concerning the probable links that can be created between data about mortality and morbidity, and the entities described in the current work. Also, gave support by highlighting the main entities which should be modified in order to adjust the representation.

ZM - This author supported the development of the ideas, discussing the main topics that should be addressed when representing mortality and morbidity. Also, gave support to the epidemiological issues which were taken into consideration in the current work.

DS - This author reviewed the manuscript several times, during the writing. He also gave ideas concerning to the content, and helped in adjusting the model. His contributions were crucial to organization of the article and the topics addressed.
